# Community Pharmacy Use by Children across Europe: A Narrative Literature Review

**DOI:** 10.3390/pharmacy6020051

**Published:** 2018-06-04

**Authors:** Mitch Blair, Arjun Menon

**Affiliations:** 1 Department of Paediatrics, Imperial College London, London SW7 2AZ, UK; m.blair@imperial.ac.uk; 2 Imperial College School of Medicine, Imperial College London, London SW7 2AZ, UK

**Keywords:** community pharmacy, pharmacist, children, parent, Europe

## Abstract

The use of community pharmacies across Europe has potential to alleviate the burden on overstretched healthcare providers. Children and young people (0–18 years) account for a large number of primary care attendances. This narrative literature review between January 2000 and December 2017 examines the use of community pharmacy by paediatric patients in Europe. The results report both positive and negative perceptions of community pharmacy by parents and children, opportunities for an extended role in Europe, as well as the need for further training. The main limitations were the inclusion of English language papers only and an initial review of the literature carried out by a single researcher. It remains to be seen whether a ‘new-look’ role of the community pharmacist is practical and in alignment with specific European Commission and national policies.

## 1. Introduction

Community pharmacy is a field of primary care medicine, which serves a large proportion of the European population. As of 2016, there were an estimated 400,000 community pharmacists in Europe visited by 46 million citizens daily [1,2], representing almost 10% of the 508 million people living in the European Union (EU) [3]. In general, the core pharmacy services across the EU include dispensing medication, medication management, emergency care (e.g., emergency contraception), as well as minor ailment management [1,2]. An example of this is in Slovakia, where up to 74% of emergency room attendances were due to lack of available alternative primary care services [4]. Further to this, in the United Kingdom (UK), the National Health Service (NHS) reported that 18 million general practitioner (GP) appointments and 2.1 million emergency department (ED) visits could have been treated at a community pharmacist and would have saved the health service £850 million in 2017 alone [5]. The paediatric population in particular have contributed to the rise in ED attendances with the UK seeing an increase in attendances of 28% from 1999 to 2010 [6]. A large part of this was attributed to the failure of other primary care services in dealing with children that had minor illnesses [6]. There is an opportunity for community pharmacists to redefine their role in the management of childhood illness. They can be the first port of call for illnesses and alleviate the burden of treating non-severe pediatric presentations in other over-stretched primary care facilities [7]. 

A number of pharmacists in Europe provide services such as routine vaccinations, chronic disease management, smoking cessation, early screening, and testing of disease [1,2]. In Belgium for example, asthma patients prescribed inhaled corticosteroids were given two follow up appointments with a community pharmacist [8]. This pilot programme was shown to benefit up to 36,000 patients, with the service potentially expanding to other chronic conditions [8]. In the UK, 1.1 million children suffer from asthma, with the NHS spending over £1 billion on the disease annually [9]. With the condition being so manageable in the community setting, it is surprising that community pharmacists do not deal with a greater number of cases to prevent hospital admissions. Another example is in Ireland where over 50,000 patients (a tenth of people vaccinated) were given the flu vaccine by community pharmacists in 2014 [1,2]. In 2015, owing to the success of the scheme, the pharmacist remit was increased to include pneumococcal and shingles vaccines as well [10]. Children are the most common recipients of such vaccines and therefore, it is important to understand why a community pharmacist does not manage more of these vaccinations for children across Europe. 

This literature review aims to understand how community pharmacy is currently being utilised by children, young people, and parents across Europe. Furthermore, it will possibly give insight into the current role of the community pharmacist in paediatric treatment, and how this role might change in the future. 

## 2. Materials and Methods

The researchers, with the assistance of the librarian, developed appropriate search parameters and identified the most relevant databases for the review, to be conducted over two weeks in December 2017. The researchers looked for data published between January 2000 and December 2017. An extensive exploratory search was performed and the literature was thoroughly assessed and synthesised in order to generate conclusions and consider future steps. 

**Figure 1:**
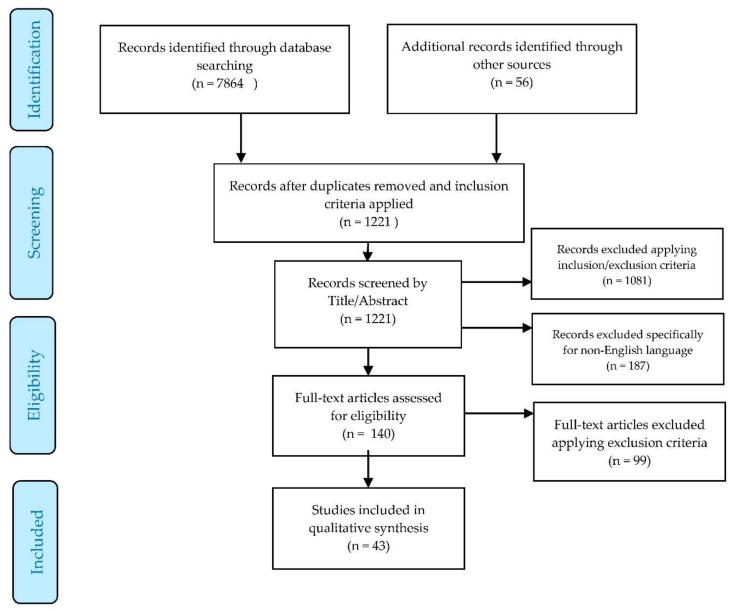
PRISMA flowchart [11] of how the literature was filtered to end up with the relevant articles for synthesis.

Electronic databases were searched including PubMed, EMBASE, OVID, and HMIC (for grey literature), with each one being thoroughly searched using as many permutations of the following key words: “community pharmacy”, “community pharmacist”, “pharmacy”, “pharmacist”, “children”, “paediatric”, “parent”, “carer”, “over-the-counter”, and “Europe”. The databases were searched by combining Boolean logic with truncation marks to generate a comprehensive set of literature. The initial literature was then subject to the specific inclusion/exclusion criteria seen in Table 1 to identify results that were in line with the objectives of the study. Once articles were obtained through database searching, the references of these articles were then screened to ensure that no relevant literature was missed from the databases. A handful of relevant articles were identified through this method, in addition to the ones found via databases. Figure 1 provides an in-depth view of how the literature was screened and selected for synthesis in the form of a narrative review, through a PRISMA flowchart [11]. It should be noted that all the studies included in the synthesis were screened individually and collectively for publication bias and selective reporting and this is reported in the results section below.

## 3. Results

Table 2 below demonstrates the number of studies from each country that were included in the synthesis.

In total, 43 studies were included in the synthesis as shown in the tables and PRISMA flowchart above. These studies demonstrated no clear publication bias or selective reporting. Non-English language studies (*n* = 187) were excluded due to translation resource restrictions. Table 3 gives an overview of all the results included, showing the type of study conducted as well as the key findings.

The results are described by theme. 

### 3.1. Perceptions

#### 3.1.1. Parental Perception

Pharmacists are regularly compared by parents, to other sources of information about medication [12]. A UK study found that 87% of the time, mothers report such advice in dealing with minor illness in children, to be ‘helpful’ in managing the problem [13]. Another stated that 82% of parents reported that they have received ‘very helpful’ or ‘helpful’ advice from pharmacists regarding non-prescription medications, while 90% of the advice given had been fully understood by parents [14]. 

However, there are still variable levels of public trust in pharmacy services. Parents prioritise health information from the Internet and GPs over and above that given by pharmacists [15]. A study in Finland showed that only 44% of parents sought advice on treating their ill children from pharmacists compared to 70% who would seek a physician’s advice [16]. If parents were to receive advice from health care professionals however, they preferred receiving written advice from a pharmacist or doctor (50%), or spoken advice from a pharmacist (29%) [17]. 

Parents identified various reasons for not trusting community pharmacist advice. In Greece, parents were hesitant to utilise pharmacists because of the lackadaisical attitude to antibiotic sale without prescription, possibly leading to wide scale resistance and adverse events [18]. A separate study in Netherlands indicated that parents did not trust explanations of doses and administration of drugs from a pharmacist, instead trusting their own limited knowledge more [19]. In addition to this, UK parents felt that pharmacists simply ‘lacked adequate knowledge’ specifically for their children [12]. 

Furthermore, the setting of the actual pharmacy may have an important effect on perception of the service. Parents felt that having substance users loitering in pharmacies, while awaiting harm reduction services and advice, was extremely intimidating to both their children and themselves [20]. This complemented the results of a study across the UK, which indicated that parents of young children found that pharmacies ‘lacked privacy’ that may otherwise be found at other primary care interfaces [21]. 

#### 3.1.2. Young People

Children and young people across Europe have their own opinions on community pharmacists. A 2014 study of over 4000 adolescents across France, Germany, Portugal, and the UK demonstrated that pharmacists are more highly trusted by this age group than any other age group [15]. This notion was further confirmed by a UK trial of pharmacy-based OCP provision [21]. Following the trial, a staggering 97% of adolescent girls reporting being ‘very comfortable’ or ‘comfortable’ in discussing contraception with a pharmacist, while 87.5% were satisfied with the service they received [21]. In fact, owing to the trusted relationship that pharmacists are likely to share with patients from repeat encounters [22], they argue that they should have an expanded role in administering HPV vaccines and giving OCP to adolescent girls [23]. 

### 3.2. Potential Opportunities

The literature identified several opportunities for pharmacists to deal with paediatric patients in various capacities. 

#### 3.2.1. Acute Minor Illness

The literature identifies the possibility of an increased role for pharmacists in dealing with acutely unwell children. One UK study showed that 9% of ED attendees were appropriate for treatment by community pharmacists instead [24]. Similarly, 15% of consultations with children attending general practices in the UK were deemed unnecessary as opposed to just 6% in adults [12]. These studies demonstrate an opportunity for the diversion of children from other primary care services to the community pharmacy.

#### 3.2.2. Chronic Disease 

Several studies highlighted the important role pharmacists could play in the management of chronic paediatric disease. Pharmacist intervention in childhood eczema was trialled in a study, resulting in a statistically significant reduction in the severity of all symptoms. Furthermore, 78% of parents found the advice given to be ‘very helpful’ or ‘helpful’, with a marked increase in effective emollient use [25]. A similar pharmacist-led intervention was trialled in Spain, with the aim of improving childhood asthma control [26]. The intervention led to an increase in quality of life measures by 0.81 points, as well as a significant reduction in poorly controlled asthma by 1.15 points compared to the control group [26]. However, in the control of type 1 diabetes in children, patients in the pharmacist-led intervention group were shown to have no significant difference in their HbA1c compared to the control group [27]. Researchers suggested that this was due to poor follow-up attendance among the intervention group [27].

In order for pharmacists to have a truly meaningful impact on the management of chronic conditions, they need to foster stronger relationships with adolescents [17]. Authors argue that if children were present at medication collection, pharmacists could then make them feel comfortable with the information being provided and the setting [17]. Other literature also agrees that regular attendance of children at medication collection could improve adherence [28]. This combined with counselling of the children and distribution of relevant leaflets, can help reduce the rate of medication non-adherence in this population group [28]. Specifically for adolescents, it was found that they could be counselled on the interaction of medications with alcohol or other drugs, as this is both relevant to their lifestyle and can help build rapport with a ‘non-judgmental’ pharmacist [14].

Furthermore, to successfully manage chronic conditions in children, more could be done to facilitate communications between the hospital, community pharmacy, and primary care in order to improve continuity of care for children and parents [29]. This is especially because of the lack of information sharing between these three interfaces of care, regarding paediatric medication [29]. To ensure that parents remain committed and not frustrated with the care of their unwell child, there must be a greater deal of cohesion between the services [29]. 

#### 3.2.3. Pregnancy and Antenatal Care 

A study in the Netherlands set up a new, pharmacist-led service for women trying to conceive, which looked to increase the awareness of folic acid use in pregnancy. Following the programme, it was seen that the prevalence of NTD almost halved in just a year while 93% of the community approved of the initiative [30].

#### 3.2.4. Pharmacovigilance

The literature identified the potential use of pharmacies for enhanced pharmacovigilance. The incidence of epilepsy in children under 18 is approximately 0.5% [31], while ADHD incidence in school-aged children is about 5% [32]. Management of these conditions involves medication that has a high rate of adverse events [33] and so there is a need here for medication monitoring especially in young children. Two separate studies in Scotland recommended wider pharmacist involvement in the UK Yellow Card scheme (YCS) for adverse events, to ensure that the proper side effect profiles of the drugs can be documented [34,35]. If uptake were to be increased in a sufficient number of pharmacists, this could be a very valuable source of adverse drug reaction reporting, as pharmacists are frequently the first port of call for medication issues [34,35]. 

Pharmacists could also improve their role in pharmacovigilance through increasing the number of medicines use reviews (MURs) performed on medications for chronic conditions. One English study found the level of pharmacists engaging in MURs to be as low as 23.7% when monitoring the experience of young children on medication for chronic conditions [36]. The importance was highlighted in a related UK trial study, which found an increase in the average asthma control by seven points when MURs and asthma counselling were routinely offered by pharmacists [37]. The increase in MURs however, would necessitate increased reimbursement and further training in order for pharmacists to be confident in carrying out such reviews [36].

#### 3.2.5. OTC Drug Use in Children

There are varying levels of OTC medicine usage across Europe. Almost 50% of Finnish children use OTC self-medication, especially for treating asthma and fevers [38]. In an Italian study of over 1.5 million children and adolescents, it was reported that a similar proportion (48%) of children received at least one drug prescription every month [39]. Germany seemed to have a lower reported use of OTC drug use with only 31.6% of 15 year olds having consumed them in the last month [40]. However, it was found that 30% of all medication purchased by children in Germany, especially sedatives, were being used without prescription [41]. The use of OTC prescriptions in German children was attributed to a variety of factors including greater maternal education, families with higher household incomes, non-immigrants, and children that had long-term illness [42]. In the UK, it was found that there were 24 million episodes of cough medication being used in children [43] even though a Cochrane review found no good evidence for or against the effectiveness of these medications [44]. Furthermore, the 59 deaths attributed to cough medicines were related to accidental exposure (22%), intentional overdose (6%), and medication error (16%) with the manner of overdose undetermined in the remaining 56% of cases [43]. Children are not being educated on the risks of overusing OTC medication with a Swedish study indicating that adolescent behaviour towards OTC was ‘careless and casual’ [45].

#### 3.2.6. Off-Label Drug Use

The literature gave insight into the use of off-label drugs by pharmacists, with a UK study showing that 40% of pharmacists had prescribed drugs in this way [46]. Pharmacists certainly had the highest level of concern (86%) among health care professionals, regarding off-label prescribing, with a majority arguing the need for urgent clinical trials to correct paediatric formulations [47]. They are willing to be flexible in prescribing appropriately for children although they mostly believed (66%) that they had a responsibility to warn parents about potential problems with off-label prescribing [46].

### 3.3. Further Training Needs

The literature reviewed the current practice of community pharmacists suggesting some possible improvements to ensure the standards of care were being met for children and young people.

#### 3.3.1. Emergency Setting

Disease or treatment specific training in community pharmacists has been shown to be effective. One such example was in a study of pharmacist perceptions to anaphylaxis. It was found that 77% of pharmacists were aware of adrenaline auto-injector use as the first line management of paediatric food allergy anaphylaxis [48], while only 7% were confident in using the device. This suggests that further training in emergency scenarios would be a useful addition to the knowledge base possessed by pharmacists. 

#### 3.3.2. Community Pharmacists as Medication Advisers

The literature described how community pharmacists were averse to using corticosteroids in the management of chronic asthma in children. Due to their own beliefs and their position as trusted medication advisers, they dissuaded parents from using corticosteroids in asthma management leading to what one author described as a nationwide ‘corticosteroid phobia’ in France [48]. Improved training of pharmacists in asthma care, especially around appropriate corticosteroid usage, is certainly needed to avoid over emphasis of the ‘dangerous’ side effects [49].

#### 3.3.3. Drug Safety

One of the issues that was evident in the literature review was the prevalence of safety incidents that occurred at community pharmacies. A study of about 3000 safety incidents in England and Wales alone revealed that medication errors occurred at a much higher rate in community pharmacies than general practice, dental surgeries, or even community nursing services [50]. Authors argued the case for a barcoding system that could reduce error potential from manual entry and act as a further safety check [50]. 

Other than technical errors in prescribing, pharmacists occasionally make mistakes in the choice of medication for acutely unwell children. A Swedish ‘mystery-shopper’ study indicated that pharmacists provided inappropriate medication to feverish children, up to 6% of the time [51] and a French study found that pharmacists were offering strictly contra-indicated drugs to children 12.9% of the time [52]. 

A Dutch study argued that these errors might be the result of a low use (14%) of up-to-date literature by pharmacists, while a large proportion of pharmacists (60%) were extremely dependent on the product monographs [53]. However, the poor choice of medication may also be explained by the history taking of pharmacists in acutely unwell children. A Belgian study showed that 84% of pharmacists did not enquire about dehydration symptoms in an eight-month-old with acute diarrhoea, while only 30% proceeded to prescribe an ORS [54]. Giving them the benefit of the doubt, some literature suggests that pharmacists’ knowledge may not be applied adequately due to a lack of questioning, poor communication skills, and parental pressures [52]. One possible solution to this is increasing the frequency, and improving the quality, of training received by pharmacists, alongside their routine practice [50]. 

## 4. Discussion

This is the first major narrative review of community pharmacy use by parents, children, and young people in Europe. Overall, parents and young people have a high regard for local community pharmacists who are considered as authoritative sources of information and advice particularly for minor illness, chronic disease management, and medication review. Issues of communication with primary care and hospital pharmacy providers remain an area for further quality improvement to ensure both timely and consistent advice as well as safe prescribing. We have found that adolescents, in particular, value the confidentiality aspects of community pharmacists especially in relation to sexual health and vaccination advice. On the other hand, the high use of OTC products and off-label drugs by this age group is of concern and presents both opportunities for health education and a greater attention to pharmacovigilance. 

Despite the fact that pharmacists may not be necessarily seen as first points of contact when compared to general practitioners or the internet from a parental perspective, there is potential for additional training and accreditation to support the needs of this age group. This would allow a broadening of the scope of services offered in a suitable physical environment. Infants and young children’s health is very much determined by the health of the mother in pregnancy and pharmacies can provide a valuable service in identification of at risk women and optimization of infant health. This could be done through ensuring nutritional supplementation throughout pregnancy like in the Netherlands where there was a substantial reduction in the incidence of neural tube defects found in the intervention group [30]. With appropriate training and accreditation, many more services could be offered by community pharmacists including increased access to smoking cessation, weight management services, and drug or alcohol reduction services. These in turn could have a considerable impact on maternal and infant health. We hope to soon report on a paediatric–pharmacy joint training initiative scheme currently being piloted in three areas of London, which might provide a useful model for other countries.

## 5. Conclusions

Community pharmacists in Europe are an untapped resource for children, young people and their parents and further recognition of their important role could usefully support the demands made on other primary care services.

## 6. Limitations

There are a few limitations of the review that may have affected the results obtained. The main limitation was that only English language studies were included in the research due to the limited resources. Following on from this, only a small number of English language research articles were published in other EU countries. Of the articles used in the synthesis, 18 were papers from the UK, while the remainder originated from 11 different countries. This represents a bias in the results, as across countries, there are likely to be national and cultural differences with regards to community pharmacy practice. Inclusion of foreign language papers may have revealed pertinent work from the other countries that may have informed future practice and training recommendations. Furthermore, the results were screened by just one researcher and therefore, some relevant results may have been excluded based on the researchers’ own selection bias of what was included in the synthesis. To mitigate this, any uncertainty was discussed by both authors who then agreed upon inclusion/exclusion. Moreover, time and resource limitations meant that the researchers were not able to obtain and confirm additional data from individual study investigators.

## 7. Further Research

This research could be used to support a periodic EU survey of parents and young people, which would ensure a more representative sampling of countries. Studies are clearly needed on the effectiveness of any pharmacy training initiatives which enhance safe prescribing practices or are designed to divert minor illnesses from emergency departments with appropriate safety netting. 

## Figures and Tables

**Table 1:** Inclusion and exclusion criteria for the literature at each stage of the research.

Inclusion Criteria	Exclusion Criteria	Justification
**At search string level:**		
Papers published between January 2000 and December 2017		The role of community pharmacists compared to present day use may be significantly different
Any article type		This allows for a comprehensive review
Full paper available		The full paper needs to be analysed in order for the review to be robust
**At title/abstract level:**		
	Paper does not specifically relate to use of community pharmacy by children	This literature review aimed to study the utilisation of community pharmacy by paediatric populations (0–18 years) in Europe
English language paper		The resources were not sufficient to fully translate papers
Europe		This literature review aimed to study the utilisation of community pharmacy by paediatric populations (0–18 years) in Europe
**At full-text level:**		
Paper analyses utilisation of community pharmacy by children		These articles are relevant to the study
	Paper only mentions community pharmacy as the location of a study	This study aims to explore the utilization of community pharmacies as a primary care service

**Table 2:** Number of studies included in synthesis stratified by country.

Country	Number of Studies Included in Synthesis
United Kingdom (England, Scotland, Wales, Northern Ireland)	19
Sweden	5
Netherlands	4
France	4
Germany	3
Finland	2
Greece	1
Iceland	1
Belgium	1
Spain	1
Italy	1
Croatia	1

**Table 3:** Description of the synthesized studies.

Reference as in Text	AuthorNo (Year)	Type of Paper/Study	Population Size	Main Study Finding
[12]	Hammond (2004)	Cross-sectional questionnaire-based study	3984	Pharmacists are a good source of information for unwell children
[13]	Hodgson (2004)	Cross-sectional questionnaire-based study	85	87% of mothers find pharmacist advice for sick children helpful
[14]	Gray (2011)	Cross-sectional mixed methods study	134 questionnaires, 39 interviews	82% of parents find pharmacist advice somewhat helpful regarding medication
[15]	Bamford (2015)	Grey literature (Charity report)	Not applicable	Parents prioritise other information sources over pharmacists
[16]	Holappa (2012)	Cross-sectional questionnaire-based study	4020	44% of parents seek pharmacist advice for their sick children
[17]	Gray (2017)	Cross-sectional mixed methods study	Not provided	Most parents prefer receiving written (50%) or spoken (29%) advice from pharmacists
[18]	Plachouras (2010)	Cross-sectional questionnaire-based study	174	Inappropriate dispensing of antibiotics by pharmacists possibly led to widespread antibiotic resistance
[19]	Stakenborg (2016)	Cross-sectional focus group-based study	24	Parents trust their own knowledge more than advice from pharmacists
[20]	Gidman (2014)	Cross-sectional focus group-based study	26	Parents did not want to bring children to pharmacies due to presence of substance misuse patients in the clinic
[21]	Parsons (2013)	Cross-sectional questionnaire-based study	99	Parents found that pharmacies lack privacy compared to other primary care services
[22]	Guegan (2010)	Literature review	Not provided	Pharmacists have a trusted relationship with young patients formed over multiple encounters
[23]	Karamanidou (2016)	Cross-sectional interview-based study	15	Expanded role for pharmacists in oral contraceptive pill (OCP) provision and HPV vaccine
[24]	Terry (2016)	Cross-sectional observational study	1623	9% of ED attendances could have been dealt with in pharmacies
[25]	Carr (2007)	Pre-post interventional pilot study	50	Effective example of pharmacist led intervention in childhood eczema
[26]	Jacome (2003)	Pre-post interventional pilot study	164	Effective example of pharmacist-led intervention in childhood asthma
[27]	Gay (2006)	Randomised control trial	100	Ineffective example of pharmacist-led intervention for Type 1 Diabetes
[28]	Koster (2015)	Cross-sectional interview-based study	170	Regular attendance of children when collecting medication can improve medication adherence
[29]	Terry (2012)	Literature review	24 references	Increasing the communication between pharmacies and other primary care interfaces
[30]	de Jong-van den berg (2008)	Explorative comparative study	Not provided	Effective example of pharmacist-led intervention in the reduction of neural tube defects (NTDs)
[31]	Deacon (2011)	Statistics report (Epilepsy)	Not applicable	Incidence of epilepsy in children
[32]	Bilbow (2016)	Grey Literature (Charity report)	Not applicable	Incidence of attention deficit hyperactivity disorder (ADHD) in children
[33]	Perucca (2005)	Literature review	55 references	Medications for ADHD and epilepsy have a high rate of adverse events
[34]	Tobaiqy (2010)	Cross-sectional questionnaire-based pilot study	72	Exploring wider pharmacist involvement in the UK Yellow Card scheme
[35]	Stewart (2005)	Prospective questionnaire-based study	267	The pharmacist as a useful resource for therapeutic drug monitoring
[36]	Aston (2017)	Cross-sectional questionnaire-based study	76	23.7% of pharmacists performed medicine use reviews (MURs) for children’s medication
[37]	Liley (2016)	Pre-post interventional pilot study	15	Increased use of MURs by pharmacists seems to improve children’s asthma control
[38]	Ylinen (2010)	Cross-sectional questionnaire-based study	4032	50% of children use over-the-counter (OTC) medication
[39]	Clavenna (2009)	Retrospective cohort study	1,542,203	48% of children receive 1 drug prescription a month
[40]	Italia (2015)	Retrospective cohort study	3013	31.6% of children used an OTC drug in the last month
[41,42]	Koelch (2008)/Du (2009)	Retrospective cohort study	17,450	30% of prescription medications for children were being used without proper prescription
[43,44]	Smith (2008)/Smith (2008)	Systematic literature review (searching for randomised controlled trials)	3492	The various causes of mortality from children using cough medicines
[45]	Holmstrom (2014)	Cross-sectional questionnaire-based study	77	Careless and casual behavior of children towards OTC use
[46]	Stewart (2007)	Prospective questionnaire-based study	482	40% of pharmacists prescribed drugs off-label
[47]	Mukkatash (2011)	Cross-sectional questionnaire-based study	563	The need for clinical trials to change paediatric formulations, to reduce off-label prescribing
[48]	Hanna (2016)	Cross-sectional questionnaire-based study	90	Only 77% of pharmacists knew to use an adrenaline auto-injector for food anaphylaxis
[49]	Raffin (2016)	Cross-sectional questionnaire-based study	500	Pharmacist perpetuate a corticosteroid phobia in the population
[50]	Rees (2017)	Cross-sectional mixed methods study	2191	Medication errors occur at a much higher rate in community pharmacies than other primary care interfaces
[51]	Bardage (2013)	Cross-sectional questionnaire-based study	1098	6% of pharmacists provided inappropriate medication to febrile children
[52]	Lapeyre-Mestre (2004)	Cross-sectional questionnaire-based study	176	12.9% of pharmacists provided contra-indicated medication to children
[53]	Venables (2015)	Cross-sectional focus group-based study	4	60% of pharmacists were very dependent on product monographs
[54]	Driesen (2009)	Cross-sectional questionnaire-based study	101	Only 30% of pharmacists prescribed oral rehydration solution (ORS) to children with severe diarrhoea
